# Role of MAOI drugs as triggers of manic episodes in bipolar disorders: A case report and a narrative review

**DOI:** 10.1192/j.eurpsy.2022.1032

**Published:** 2022-09-01

**Authors:** L. Olivier Mayorga, L. Ilzarbe, H. Andreu Gracia, L. Bueno Sanya, O. De Juan Viladegut, P. Barrio

**Affiliations:** Hospital Clinic de Barcelona, Department Of Psychiatry And Psychology, Institute Of Neuroscience, Barcelona, Spain

**Keywords:** treatment-emergent affective switch, TEAS, bipolar disorder, MAOI

## Abstract

**Introduction:**

Use of Monoamine Oxidase Inhibitors (MAOIs) has experimented an important reduction in recent years, being replaced by other antidepressant drugs (ADs) associated with a better safety profile. Its use has been restricted to instructed professionals treating resistant and atypical depression. Thus, treatment-emergent affective switch (TEAS) induced by MAOIs is a rare event nowadays.

**Objectives:**

To describe a manic episode associated to a one-year-long treatment with phenelzine, a MAOI agent.

**Methods:**

We present the case of a 47-year-old man hospitalized in our acute psychiatric unit after presenting compatible clinical symptoms with a manic episode. He showed severe irritability, decreased need for sleep, pressured speech, increased energy and goal-directed activities. The patient had started phenelzine a year ago for the treatment of major depressive episode resistant to previous pharmacological essayed treatments. No previous history of TEAS was reported, although he had already taken other ADs and mood-stabilizer treatments in the past.

**Results:**

Several studies reported the effectiveness of MAOIs for the treatment of monopolar depressive episodes resistant to other ADs, especially when atypical symptoms were observed. Data on the use of MAOIs for the treatment of drug-resistant bipolar depressive episodes is scarce. Few studies have described a good response without showing and increased risk of TEAS.

**Conclusions:**

As MAOIs have fallen out of favour with modern psychiatry, there is scarce evidence on the prevalence of TEAS in patients undergoing treatment with these drugs. Further research is needed in order to accurately define these complex relationships.

**Disclosure:**

No significant relationships.

